# A paradigm shift in neutrophil adverse event grading: Why now?

**DOI:** 10.1002/hem3.70242

**Published:** 2025-10-27

**Authors:** Lauren E. Merz

**Affiliations:** ^1^ Department of Medicine Division of Hematology/Oncology University of Michigan Ann Arbor Michigan United States

The Common Terminology Criteria for Adverse Events (CTCAE) is a comprehensive list of laboratory and clinical findings that could represent toxicity or adverse events (AEs) associated with systemic anticancer therapy use. These criteria are used by most cancer clinical trials globally and are very useful in ensuring consistent reporting of AEs and comparison of toxicities between trials. The CTCAE also provides grades to reflect severity. Grade 1 indicates mild to asymptomatic events, Grade 2 represents moderate events with noninvasive interventions potentially required, Grade 3 highlights severe or medically significant events, Grade 4 represents life‐threatening events, and Grade 5 indicates death from the AE.^1^ These lists are released by the National Cancer Institute (NCI) in the United States and updated periodically—typically to improve clarity, update terminology, or reflect advances in therapy. There is no clear temporal pattern to these updates. Since 1982, six versions of the CTCAE criteria have been released.^1^ The most recent (v6) was released in the summer of 2025, with planned implementation for clinical trials on January 1, 2026.

One of the most significant changes is the update to the grading of neutrophil counts (Table 1). This criterion is very commonly used in cancer clinical trials and has never been changed before. CTCAE v6 functionally translates neutropenia grade up by one level, where <1500 to 1000/µL is now Grade 1 (previously Grade 2). Grade 4 is now absolute neutrophil count (ANC) < 100/µL. CTCAE v1–5 Grade 1 neutropenia (lower limit of normal [LLN] to 1500/µL) no longer exists. Neutropenia grades are intended to correlate with the risk of life‐threatening infections and complications like febrile neutropenia. However, in the past 40 years since the neutrophil grades were initially established, we have discovered that the ANCs that are associated with a medically significant event or death differ with genetic variants,^2^ type of systemic anticancer therapy administered,^3^ and both the duration and the degree of neutropenia.^4^

**Table 1:** Comparison of neutrophil count Common Terminology Criteria for Adverse Events (CTCAE) grading criteria between v1–5 and v6.

	CTCAE v5 (2017)	CTCAE v6 (2025)
Grade 1	LLN to 1500/µL	<1500 to 1000/µL
Grade 2	<1500 to 1000/µL	<1000 to 500/µL
Grade 3	<1000 to 500/µL	<500 to 100/µL
Grade 4	<500/µL	<100/µL

*Note*: The shading highlights the updates to ANC levels of the grading criteria between the different CTCAE versions.

Abbreviation: LLN, lower limit of normal.

The CTCAE v6 updates are timely and necessary. Although the rationale behind the changes to CTCAE v6 neutrophil count criteria is not provided, there are likely two major reasons for the updates to the neutropenia criterion: inclusion of people with the Duffy null variant and acknowledgment of modern therapy impacts on neutrophil physiology.

The Duffy antigen is a protein found on erythrocytes.^5^ The null form is partially protective against infection with *Plasmodium vivax* and thus is commonly seen in people with genetic ancestry from the African continent and the Arabian Peninsula.^5^ In fact, 80%–100% of people living in Western Africa, ~80% of people identifying as African or Afro‐Caribbean in the United Kingdom, and 66% of people identifying as Black or African American in the United States have the Duffy null phenotype.^2^, ^6^, ^7^ This variant also results in significantly lower circulating neutrophil counts due to preferential localization in the spleen (rather than the periphery), without an increased risk of infection.^8^ The normal ANC reference interval for Duffy null adults is 1200–1540/µL compared to 2000–7500/µL for Duffy non‐null adults.^9^

As the LLN ANC for Duffy null adults is 1200/µL, approximately 25% of Duffy null adults had Grade 1 (2000–1500/µL) or Grade 2 (<1500 to 1000/µL) neutropenia by CTCAE v1–5 at their healthy baseline.^2^, ^9^ With the CTCAE v6 update, very few Duffy null adults are expected to have Grade 2 (<1000–500/µL) neutropenia, and <10% are expected to have Grade 1 (1500–1000/µL) neutropenia at baseline.^2^, ^9^ This is a significant improvement in accurately reporting toxicity in diverse, global populations that will help individual patients remain on trial and receive systemic anticancer therapy at optimal doses as well as encourage trialists to recruit and retain diverse populations without fear of excess neutropenia AE.^10^

Furthermore, CTCAE grading is often linked to eligibility criteria. For example, a very common ANC threshold for trial eligibility is ≥1500/µL (CTCAE v1–5 Grade 2; CTCAE v6 Grade 1).^11^ A recent study of real‐world patients highlighted that Duffy null patients have ~10% lower eligibility for clinical trials due to ANC criteria alone.^12^ In fact, there are significant differences in eligibility by Duffy status due to ANC alone at all ANC thresholds ≥1100/µL.^12^ As the Duffy null variant predominantly impacts non‐White patients, this is certainly a contributor to racial disparities in clinical trial participation.^10^ If ANC eligibility thresholds remain linked to CTCAE criteria, these updates should naturally result in future clinical trials using an ANC threshold of ≥1000 or lower (CTCAE v6 Grade 2), which will significantly ameliorate differences in eligibility by Duffy status due to ANC.

In addition to a growing awareness of the Duffy null variant and a desire to be as accurate and inclusive as possible, the CTCAE v6 updates may also reflect the mechanism of neutropenia of most modern systemic anticancer therapies and associated risk of infection. Historically, cancer therapies were very cytotoxic and even myeloablative. This resulted in low to no reserve of neutrophils in human bodies that require 7–10 days to develop a mature neutrophil from a hematopoietic stem cell, which resulted in a high risk of infection when ANC was <500/µL.^13^, ^14^ However, many modern therapies are targeted, and the mechanism of cytotoxicity (if any) is different from those of older therapies. For example, CDK 4/6 inhibitors have a neutropenia AE incidence of ~80%, with two‐thirds of patients experiencing CTCAE v1–5 Grade 3 and 4 neutropenia that dictates dose modification.^15^ However, there are very low rates (<2%) of febrile neutropenia.^15^ This level of disconnect between neutropenia AE and febrile neutropenia is not seen in traditional cytotoxic protocols. These differences are likely because the mechanism of neutropenia with CDK 4/6 inhibitors is maturation arrest rather than destruction resulting in partially preserved function and rapid recovery.^15^ Thus, it is no longer accurate or appropriate to use neutropenia severity grading from the 1980s to assess treatments from the 2020s. The CTCAE v6 neutropenia grading update incorporates 40+ years of assessing association of ANC with complications as well as the realities of modern therapies to better reflect degree of concern for febrile neutropenia or serious infectious complication, with Grade 3 (ANC < 500–100) highlighting severe or medically significant events and Grade 4 (<100/µL) representing life‐threatening events.

We applaud the NCI and CTCAE committee for this timely and necessary update to the neutrophil AE criteria. This is an appropriate adjustment that accounts for variation in normal neutrophil counts driven by the Duffy null variant and more accurately quantifies toxicity and degree of concern with modern therapies. The question remains: “What now?”. How will this impact ongoing and future clinical trials—particularly around eligibility criteria and dose modifications that are historically linked to CTCAE grades? Will this impact ANC criteria used for standard of care protocols? How can we use these updates to reflect on our current or future clinical trial protocols to ensure that our parameters are necessary and personalized to the populations that we serve and the drug that we are using? The CTCAE v6 neutrophil grading update is an exciting and critical change that will affect nearly every clinical trial, but the magnitude and reach of these updates on clinical trial parameters and systemic anticancer therapy administration are yet to be seen.

## AUTHOR CONTRIBUTIONS

**Lauren E. Merz**: Conceptualization; writing—original draft; writing—review and editing.

## CONFLICT OF INTEREST STATEMENT

Dr. Merz reports receiving personal fees from Johnson & Johnson and 23andMe.

## FUNDING

This research received no funding.

## Data Availability

Data sharing is not applicable to this article as no data sets were generated or analyzed during the current study.
